# Supplementary material for: Molecular and Phylogenomic Analysis of a Vancomycin Intermediate Resistance USA300LV Strain in Chile
Source: Microorganisms. 2024 Jun 25;12(7):1284. doi: 10.3390/microorganisms12071284 (PMC11278659; doi:10.3390/microorganisms12071284)
Supplement: Supplementary file 1 [file microorganisms-12-01284-s001.zip › microorganisms-3032996-supplementary-initial-file.pdf]

microorganisms

Supplementary Materials

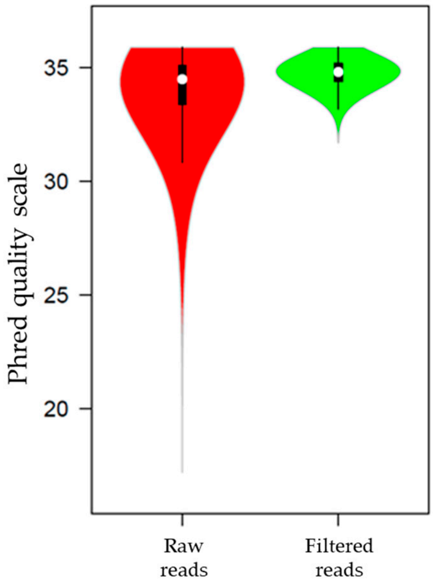

Figure S1. Graphic representation of isolate n°42 reads quality improvement after quality control. Representation was made considering the first 500 thousand reads.

Table S1. Primers used for the detection of virulence factors.

Gene	Primer Sequences (5′->3′)	Reference
pvl	F: ATCATTAGGTAAAATGTCTGGACATGATCCA<br>R: GCATCAAATGTATTGGATAGCAAAAGC	[18]
tsst	F: TTCACTATTTGTAAAAGTGTCAGACCCACT<br>R: TACTAATGAATTTTTTTATCGTAAGCCCTT	[18]
etA	F: ACTGTAGGAGCTAGTGCATTTGT<br>R: TGGATACTTTTGTCTATCTTTTTCATCAAC	[18]
sdrC	F: ACGACTATTAAACCAAGAAC<br>R: GTACTTGAAATAAGCGGTTG	[22]
sdrD	F: GGAAATAAAGTTGAAGTTTC<br>R: ACTTTGTCATCAACTGTAAT	[22]

Table S2. COGs annotation categories.

Character	COG
D	Cell cycle control, division, chromosome partitioning
M	Cell wall/membrane/envelope biogenesis
N	Cell motility
O	Post-translational modification, protein turnover, chaperones
T	Signal transduction mechanism
U	Intracellular trafficking, secretion, and vesicular transport
V	Defense mechanism
W	Extracellular structures
Y	Nuclear structure
Z	Cytoskeleton
A	RNA processing and modification
B	Chromatin structure and dynamics
J	Translation, ribosomal structure, and biogenesis

K	Transcription
L	Replication, recombination, and repair
X	Mobilome: prophages, transposons
C	Energy production and conversion
E	Amino acid transport and metabolism
F	Nucleotide transport and metabolism
G	Carbohydrate transport and metabolism

Table S3. Alignment of element COMER components between *S. aureus* CA12 strain and isolate n°42.

**Protein**	**Orientation**	**Identity percentage**
abi-a	Positive	100
abi-c abortive phage resistance protein	Positive	99,9
TR DNA-binding protein	Positive	100
abi CAAX protease	Positive	100
MutS DNA mismatch repair protein	Positive	100
HP	Positive	99,9
membrane protein	Negative	100
NMO nitronate monooxygenase	Negative	100
LysR like	Negative	100
MFS tp	Negative	99,9
transposase	Positive	100
3-demethylubiquinone-9 3-methyltransferase	Negative	100
trans	Positive	100
pyrox pyridine nucleotide-disulfide oxidoreductase	Positive	100
merR	Positive	100
HP	Positive	100
cytochrome c	Positive	100
mtp mercury transporter	Positive	100
merA mercuric reductase	Positive	100
merB alkylmercury lyase	Positive	100
HP	Negative	100
HP	Positive	100
HP	Positive	99,6
integrase	Negative	100
copB ATPase	Positive	100
mco	Positive	100
Lipoprotein putative transporter YdhK	Positive	99,8
